# Dynamic Modelling and Simulation of Food Systems: Recent Trends and Applications

**DOI:** 10.3390/foods12030557

**Published:** 2023-01-27

**Authors:** Jose A. Egea, Míriam R. García, Carlos Vilas

**Affiliations:** 1 Fruit Breeding Group, CEBAS-CSIC, Campus de Espinardo 25, 30100 Murcia, Spain; 2 Biosystems and Bioprocess Engineering Group, IIM-CSIC, 36208 Vigo, Spain

Several factors influence consumers’ choices of food products. While price remains the main criterion, quality, pleasure, convenience, and health are also important driving factors in food market evolution. Food enterprises are making significant efforts to manufacture products that meet consumers’ demands without compromising on safety standards. Additionally, the food industry also aims to improve the efficiency of transformation and conservation processes by minimizing energy consumption, process duration, and waste generation. However, foods are highly complex systems in which: (i) non-linear dynamics and interactions among different temporal and spatial scales must be considered; (ii) a wide range of physical phenomena (such as evaporation, mechanical changes, thawing, energy/mass transport, and color changes) occur; (iii) different food matrices (such as meat, vegetables, cereal, milk, and juices) with different microstructures and properties are involved; and (iv) the number of quality and safety indicators (such as bacteria, total volatile basic nitrogen, color, texture, odor, and sensory characteristics) is substantial. Mathematical modeling and simulation are key elements that allow us to gain a deeper understanding of food processes and enable the use of tools such as optimization and real-time control to improve their efficiency. This special issue aims to gather research on the development of dynamic mathematical models that describe the relevant factors in food processes from the perspectives of food safety (chemical or microbiological), food quality (organoleptic or nutritional), or resource consumption. Additionally, the development of model-based tools to improve food processes is also considered. This includes decision-making and optimization tools, the characterization of uncertainty/variability in model predictions, model simulation techniques, software sensors, and software development. The contributions published in this Special Issue can be grouped into two categories according to their main research topic: the evolution of safety and quality indicators in unprocessed food systems, and transformation and preservation processes.

## 1. Evolution of Safety and Quality Indicators in Unprocessed Food Systems

The evolution of quality in food products is mainly dependent on microbial content, but also on other indicators such as nucleotide degradation; the formation of volatile nitrogenous bases or biogenic amines; and texture. In this Special Issue, we present four research articles on different aspects of bacterial growth or inactivation and a review paper analyzing the mathematical models in the literature that describe and predict food quality indicators.

### 1.1. Bacterial Dynamics

Microbial growth and inactivation rates are highly influenced by the food matrix. Therefore, matrix microstructure is a main factor to consider when deriving mathematical models that describe microbial dynamics in food systems. Verheyen and Van Impe [1] provide a comprehensive review of the models developed during the last two decades that study microstructure influence. Two types of model are identified: (i) macroscale secondary models including food microstructural factors, and (ii) microscale semi-mechanistic models. The selection of the best approach depends on the particular application, the accuracy required, and the available computational power. The authors also identified current research trends: (i) the study of the effect of intrinsic factors on microbial dynamics, and (ii) the development of models considering the influence of food microstructure during non-thermal processes.

Some bacteria, such as *Carnobacterium maltaromaticum* CNCM I-3298, can be used in food biopreservation, flavor development processes, or in biological time–temperature integrators to track temperature variations during transport and storage. Puentes et al. [2] used the reaction scheme mechanism to derive an accurate mathematical model that describes the growth of *C. maltaromaticum* and the production of formic acid, acetic acid, lactic acid, and ethanol from trehalose. The surface response method was used to describe the relationships between the operating conditions (temperature and pH) and the specific growth and production rates. The authors also illustrated how the model can be used to compute the optimal operating conditions of the process (T and pH). Finally, they also proposed some interesting research directions such as incorporating the effects of other culture parameters or understanding the inhibition mechanisms of metabolites.

The efficiency of treatments to inactivate bacteria can be assessed by detecting and quantifying the sublethal injury of pathogenic microorganisms. However, existing methods of modeling the evolution of sublethal injury (SI) present several disadvantages related to the frequent occurrence of SI trends in these methods, which are, in part, artifacts. Akkermans et al. [3] proposed a new approach to modeling the evolution of SI during microbial inactivation that avoids unrealistic calculations. The method, based on the description of inactivation kinetics between subpopulations of healthy, sublethally injured, and dead cells, was designed to be used in combination with any existing microbial inactivation model. Log-linear inactivation, biphasic inactivation, and log-linear inactivation with tailing were used to validate the approach. The advantages of this approach make it suitable for describing SI during food processes. 

*Shewanella putrefaciens* is one of the most important Specific Spoilage Organisms (SSOs) in fish products. Yi and Xie [4] focused on designing a nondestructive method, based on the use of an electronic nose, to describe the growth of *S. putrefaciens* during fish spoilage. Bacterial concentration was described using two classical primary models—Gompertz and logistic—whereas the dependence of growth rate and lag time on temperature was modeled using the square root model. The authors also derived a regression model based on the partial least squares method to correlate the electronic nose and electrical conductivity measurements with the spoilage potential of *S. putrefaciens*. Finally, gas chromatography/mass spectrometry was used to determine the characteristic volatile organic compounds of tuna inoculated with *S. putrefaciens*.

### 1.2. Other Quality Indicators

The development of methods to describe the evolution of other quality indicators has gained relevance in recent decades. García et al. [5] presented a comprehensive review of the different indicators used in the literature to assess the quality of fresh fish; the stress variables that affect the evolution of such indicators; and the mathematical models available to describe such evolution. The work also presented the main challenges currently faced in food quality modeling:

(i) There is a lack of mathematical models for some critical indicators, such as nutrients and odor.
(ii) There are many different model structures but a lack of proper comparisons between alternatives.
(iii) Uncertainty analysis of model parameters and bacterial load is usually missed.
(iv) Model validation is usually disregarded.
(v) The relationships between the shelf life and growth of SSOs are not well understood and are usually not described in dynamic modeling.
(vi) The potential of current models is not fully exploited towards their integration into software systems for online quality prediction.

## 2. Preservation and Transformation Processes

Preservation and transformation processes are of paramount importance in food systems. This Special Issue includes four articles focusing on different aspects of the fermentation process and three manuscripts that consider thermal processes.

### 2.1. Fermentation Processes

Mathematical models are useful tools to understand food systems, and combined with proper methodologies such as optimization, control, or scheduling, they enable the design of food processes and their operating conditions. Ritonja et al. [6] derived a fourth-order non-linear state-space model to explain the effect of temperature on the dynamics of CO_2_ produced during milk fermentation. The structure of the proposed model is compact and simpler than other options in the literature, although it is able to represent experimental behavior. The authors also suggest that a non-linear adaptive control approach would be a reliable option to design a control law to force the process to follow the desired reference trajectory.

Fermentation is also used to ensure the safety and quality of foods, and to increase product shelf life. Predictive microbiology can be exploited to describe the growth and inactivation of bacteria as a function of the fermentation conditions. Racioppo et al. [7] used the Food Spoilage and Safety Predictor to model the effects of stress variables (such as temperature, pH, and salt) on the growth of lactic acid bacteria in fermented smoked fish. The maximal growth rate and the time taken to attain the critical threshold were modeled through a multiple regression procedure. This model was used to optimize the production of smoked fermented fish by combining the variables through a fractional design of experiments. The authors showed that the most critical factor in the fermentation process was liquid smoke, followed by temperature and salt.

Rapaport et al. [8] proposed a simple model that includes a maintenance term (giving rise to a variable yield) to describe the growth of yeast on nitrogen during the fermentation of wine. This maintenance term can explain a consumption of nitrogen that is not entirely converted into biomass. Additionally, the variable yield, that can be estimated from data, gives the approach the flexibility to suit different kinds of models or experimental data with a single common structure. The maintenance term encodes the underlying mechanisms of transporters and carbohydrate accumulation. The authors showed that this simple model can reproduce the experimental data and results of more sophisticated models, bringing new perspectives to the control of wine fermentation through the addition of nitrogen.

Dynamic models describing food processes must be accurate and reliable, but they must also be compatible with measurable variables in real industrial processes. Zamudio-Lara et al. [9] proposed two dynamic models of beer fermentation and performed parameter estimation, structural identifiability analysis, observability analysis, and cross-validation to assess the models’ predictive capabilities. The proposed models were based on biomass dynamics and CO_2_. A set of variables that should be monitored for each model to achieve complete observability was provided. The estimation procedure included some mathematical relationships to describe the thermal dependence of the kinetic parameters proposed, leading to a good prediction of the experimental data for both models. These new models enable measurement implementations in order to identify and quantify the process variables, thus improving process efficiency and controllability. These new models are good candidates for model-based process control in beer fermentation.

### 2.2. Thermal Processes

The analysis of multiple objectives is crucial when designing dynamic food processes. The different dynamics of the considered objectives may lead to counterintuitive conclusions. Peñalver-Soto et al. [10], the authors analyzed the microbial inactivation of *Geobacillus stearothermophilus* and acrylamide production in the thermal processing of pureed potato and prune juice, which may be present in infant formulations. The authors found that to ensure proper microbial inactivation and reduce acrylamide formation, high-temperature processes (with a short application time) are needed. This could be counterintuitive as acrylamide formation increases along with temperature. However, the sensitivities of the objectives to the process variables make the dynamics of acrylamide formation much slower than those of microbial inactivation at high temperatures. These results may facilitate the design of microbial inactivation thermal processes where acrylamide formation is an issue.

Quality parameters can be seriously affected when dynamic thermal processes are applied to foods. In some cases, high temperatures can produce a decline in some quality parameters while improving others. This is the case with fried potato chips, where higher temperatures improve yellowness and crunchiness (important indicators for consumer acceptance) but also accelerate the production of certain toxic compounds such as acrylamide. Peñalver-Soto et al. [11] presented a multi-objective optimization approach to simultaneously maximize yellowness and minimize acrylamide production in the potato-chip frying process. Their results showed that most of the solutions of the Pareto front led to levels of acrylamide above the maximum recommended by the European Food Safety Agency (EFSA). Low temperatures and high processing times should be used to avoid excess acrylamide. They also found that under mild processing conditions, there can be quasi-equivalent solutions (e.g., different processing conditions leading to the same relationship between yellowness and acrylamide) due to the sensitivities of the objectives to such conditions. Finally, parameter uncertainty and Pareto front uncertainties were higher at higher temperatures.

Innovations in the field of rapid heating technologies require foods’ thermal properties to be determined accurately. Muniandy et al. [12] performed a study to determine the thermal conductivity of a model food using rapid heating. Two-dimensional heat transfer models based on finite differences were formulated, and experiments to monitor temperature were designed based on scaled sensitivity coefficients. The authors proposed three models for thermal conductivity—constant, linear, and re-parameterized linear—to improve identifiability, and obtained better estimates from the linear ones. To estimate the parameters with low errors, it was concluded that the constant temperature experiment should be conducted for at least 20 min, while the rapid heating experiment required only 30 s. The estimated trend of conductivity with temperature was more consistent with fatty foods in the rapid heating experiments. Additionally, the residual analysis for both types of experiment revealed that the parameter estimation in the rapid heating experiment was more reliable. Finally, prolonged exposure to temperature in the constant-temperature experiments could negatively impact the reliability of the estimated thermal properties due to changes in the food matrix. 

In the face of climate change, it may advised that unused species of some crops should be recovered to ensure resistance against increasing pests and resilience against changing climate conditions. It is therefore important to determine their physicochemical properties and understand how they are affected by processing treatments.Sridhar et al. [13] determined the physicochemical properties of currant tomato (*Solanum pimpinellifolium*) and studied the effects of cold- and hot-break heat treatments on it. Color-related parameter values decreased significantly under all of the heat treatments. The apparent viscosity, lycopene, and total titratable acidity differed significantly between heat treatments (mostly at the highest temperatures). The change in the viscosity of tomato pulp and paste with temperature was modeled using Arrhenius. The findings of this research may strengthen the knowledge of process optimization designers, and thus, facilitate the development of currant tomato-based products.

## Data Availability

No data were created or used in this research.
